# Impact of systemic steroids on asthma biomarkers: A comprehensive analysis^☆^

**DOI:** 10.1016/j.waojou.2026.101363

**Published:** 2026-03-27

**Authors:** Giovanni Paoletti, Giovanni Costanzo, Marta Marchetti, Guido Valentini, Alfredo Scardini, Giada Sambugaro, Martina Bullita, Sofia Vassallo, Andrea Giovanni Ledda, Davide Firinu, Giorgio Walter Canonica, Stefano Del Giacco, Enrico Heffler, Giulia Anna Maria Luigia Costanzo

**Affiliations:** a Department of Biomedical Sciences, Humanitas University, 20072 Pieve Emanuele, Italy; b Personalized Medicine, Asthma and Allergy, IRCCS Humanitas Research Hospital, 20089 Rozzano, Italy; c Department of Medical Sciences and Public Health, University of Cagliari, 09124 Monserrato, Italy

**Keywords:** Biomarker, Steroid, Asthma

## Abstract

Asthma is a chronic inflammatory airway disease characterized by variable airflow obstruction and bronchial hyperresponsiveness, affecting millions worldwide and showing heterogeneity in its endotype, clinical presentation, and severity. Following the principles of modern precision medicine, studying an asthmatic patient involves collecting various biomarkers that may assist in diagnosis, management, and characterization of the disease to predict clinical outcomes and treatment responses. Although systemic corticosteroid use should be limited to specific situations, this therapy still plays a crucial role in managing an asthmatic patient, especially during exacerbations. While it is known that steroids can affect biomarkers, the extent of this impact is often unclear. This remains an open issue, particularly in patients with severe asthma, where systemic steroids are more frequently used and biomarker measurement can deeply influence management and even access to therapy. Misinterpreting biomarkers can lead to over- or underestimating asthma severity, potentially resulting in serious consequences. In this comprehensive analysis, we have collected the available literature data on how systemic corticosteroids can affect different biomarkers used in clinical practice and research setting, categorizing them into humoral (eg, blood eosinophil count, total and specific IgE, alfa-1 antitrypsin, interleukins, eosinophil cationic protein, and thymic stromal lymphopoietin), instrumental (eg, spirometry, impulse oscillometry, fractional and multiple flow exhaled nitric oxide, and nasal nitric oxide), and clinical (ear-nose-throat and gastroenterological evaluation). The goal is to provide a practical and concise overview of this impact to assist clinicians in interpreting biomarkers independently of steroid therapy. Research should focus on developing predictive models that adjust individual biomarker readings for steroid dosage and duration, as well as markers less affected by corticosteroid interference. A detailed understanding of steroid-induced biomarker modulation is crucial for accurate asthma phenotyping, personalized treatment choices, and avoiding adverse outcomes caused by both under- and overtreatment. Going forward, integrating improved biomarker correction methods, enhancing patient education on steroid use, and continuing research into new inflammatory markers will be crucial for improving the accuracy and effectiveness of asthma management.

## Introduction

Asthma is a chronic inflammatory disease of the airways characterised by variable airflow obstruction and bronchial hyperresponsiveness. It affects millions of individuals worldwide and is increasingly recognized as a heterogeneous condition involving a complex interplay of inflammatory and non-inflammatory mechanisms.^1^

The heterogeneity observed in asthma presentation is increasingly attributed to variations in the underlying pathobiology, highlighting the necessity for a comprehensive phenotypic and endotypic characterisation of the disease.^1^, ^2^, ^3^, ^4^

The process of phenotyping asthma has undergone significant evolution over recent decades. Initially, asthma was classified simply as either intrinsic or extrinsic based on clinical observations. However, with advancements in molecular biology and immunopathology, it has become clear that a more nuanced approach is necessary. Modern phenotyping strategies involve detailed assessments of clinical features, biomarkers, and functional parameters, enabling clinicians to delineate specific asthma subtypes. These include the well-recognized Th2-high and Th2-low endotypes, which are characterised by distinct inflammatory profiles and differential responses to therapy.^5^, ^6^ Longitudinal follow-up and advanced imaging techniques enhance our understanding of disease progression and treatment response, facilitating a more personalized approach to management. This stratification not only informs therapeutic decisions but also enhances prognostication in clinical practice.^7^

Systemic corticosteroids, particularly the oral formulations, have long been a cornerstone in the management of severe asthma and acute exacerbations. Despite their undeniable effectiveness in reducing airway inflammation and preventing life-threatening exacerbations, systemic steroids possess a double-edged nature. Their usage is frequently accompanied by a broad range of adverse effects, encompassing metabolic disturbances and immunosuppression. Notably, there is rising concern regarding the overuse of systemic corticosteroids, which may often go unreported by patients due to fears of stigma or side effects.^8^, ^9^, ^10^

This unnoticed overuse can significantly skew the interpretation of asthma biomarkers, resulting in what might be described as a “cosmetic” alteration of laboratory and instrumental findings.^11^ In many cases, systemic steroids can transiently normalize biomarkers such as blood eosinophil counts,^12^ fractional exhaled nitric oxide (FeNO),^13^ and imaging findings, masking the underlying inflammatory state.^14^ As a result, clinicians may be misled regarding the actual severity or activity of the disease, which poses challenges in both diagnosis and long-term management.

The phenomenon of biomarker modification by systemic steroids necessitates a critical evaluation of the tools available for assessing asthma severity. Emerging evidence suggests that a multimodal approach, integrating clinical assessments with humoral and instrumental biomarkers, can provide a more accurate picture of disease activity. For example, while serum markers such as periostin^15^, ^16^ and serum immunoglobulin E (IgE) levels may be altered by steroid therapy, advanced imaging techniques and functional respiratory tests could offer additional insights into residual airway inflammation and remodeling.^17^

This comprehensive analysis aims to explore the impact of systemic steroids on various asthma biomarkers, including humoral, instrumental, and clinical parameters. Specifically, this work intends to assess whether the “cosmetic” changes induced by systemic steroid use can lead to misinterpretation of disease status. By integrating data from clinical studies and real-world observations, we seek to delineate a clearer picture of how systemic steroids affect biomarker profiles and, ultimately, propose strategies to improve asthma phenotyping and follow-up accuracy in patients undergoing steroid therapy. This analysis is positioned at the intersection of clinical practice and translational research. It recognizes the complexity of asthma as a multifaceted disease influenced by intrinsic factors and external therapeutic interventions. Focusing on the underappreciated phenomenon of steroid-induced biomarker modulation, this study aims to contribute to a more refined and individualized approach to asthma management, with the goal of optimizing patient outcomes while minimizing the risks associated with systemic steroid overuse.

## Methods

We performed a narrative review of the literature. The search for articles used combinations of keywords, including terms like asthma, asthma biomarkers, systemic corticosteroids, asthma treatment. We included articles which: (1) evaluated asthma biomarkers and (2) examined they are affected by the treatment with systemic corticosteroids.

All studies published until June 2025 were deemed eligible for inclusion. The review included review articles, clinical trials, and editorials. The review was restricted to peer-reviewed literature. Grey literature, conference abstracts, and non-peer-reviewed publications were omitted to maintain methodological rigour and comparability across the included studies.

Eligibility was restricted to full-text studies written in English and published in their final form. No restrictions were imposed on the nationalities of authors or the sources of funding. The searches were performed from April 1 to June 1, 2025.

### Humoral biomarkers

The OCS's impact on humoral biomarkers will be reviewed in the following paragraphs, divided into biomarkers used in clinical practice and research settings. The data collected are summarized in Table 1 and Table 2, respectively.

**Table 1:** Clinical practice biomarkers and relationship with systemic corticosteroid therapy. References in the text. BEC: blood eosinophil count. IgE: immunoglobulin E. OCS: oral corticosteroid

Biomarker	Role in clinical practice	Altered by OCS Short-treatment	Altered by OCS Long-treatment	Corrective factors	Wash out time
**BEC**	Biomarker of T2 inflammation (phenotype)	40 mg OCS/day for 14 days reduces BEC by 76% (median) and by 93% in corticosteroid-naive patients, −41% if the maintenance dose is 10 mg.	5–35 mg/day OCS for more than 8 weeks up to 6 months of pretreatment reduces BEC by −12.	Treatment for more than 6 months with 5–35 mg/day OCS: In 6–8 weeks, reduction dose by 1 mg/day, increase 7% BEC. And reduction dose by 5 mg/day increases 41% BEC.	For short treatment (3 weeks): 79 days of washout. Unknown for longer treatment.
**Total IgE**	Biomarker of T2 inflammation (phenotype) Alternative diagnosis (ABPA) Omalizumab dosage optimization.	7 days of treatment with 20 mg OCS led to a transient increase of total IgE by 46% in 1 study.	No modification if the treatment is longer than 6 weeks	Not known	Not known for short treatment. For long-term treatment is not necessary
**Specific-IgE** **Serum levels of allergen extracts**	Analyze the type of sensitization and diagnostic support	7 days of treatment with 20 mg OCS maybe determine a polyclonal response with a transient increase (not quantified). Other studies say no modification. More studies needed.	Not known	Not known	Not known
**Precipitins**	They assist in the diagnosis of ABPA in asthma patients	Lower levels of nonspecific precipitins	Not known	Not known	Not known

**Table 2:** Clinical research biomarkers and relationship with systemic corticosteroid therapy. CCL: Chemokine (C–C motif) ligand. ECP: eosinophil cationic protein. DPP4: Dipeptidyl peptidase-4. IL: interleukin. TARC: Thymus and activation-regulated chemokine. TSLP: thymic stromal lymphopoietin

Biomarker	Clinical relevance	Altered by OCS	Corrective factors	Wash out time
**IL-4**	Controversial	Unclear	Not known	Not known
**IL-5**	Controversial	Likely decreased	Not known	60 days
**IL-13**	Controversial	Decreased	Not known	60 days
**IL-25**	Controversial	Not known	Not known	Not known
**IL-33**	Controversial	Not known	Not known	Not known
**TSLP**	Controversial	Not known	Not known	Not known
**CCL-26**	Controversial	Not known	Not known	Not known
**CCL-17/TARC**	Controversial	Decreased	Not known	Not known
**DPP4**	Controversial	Decreased by inhalatory corticosteroids	Not known	Not known
**ECP**	Controversial	Decreased	Not known	Not known
**Periostin**	Controversial	Decreased	Not known	Not known

#### Clinical practice

##### Blood eosinophil count (BEC)

Blood eosinophil count (BEC) is an essential biomarker used to classify severe asthma. Severe asthma with BEC counts greater than 300 cells/μL is suggested to be representative of underlying type 2 inflammation (T2-high endotype).^18^

Oral corticosteroid (OCS) dose is inversely correlated to BEC in patients with severe eosinophilic asthma, but there is limited data to quantify this relationship.

A meta-analysis revealed that treatment with OCS (prednisolone, methylprednisolone, or dexamethasone) for a median period of 2 weeks was associated with a reduction in BEC of about 76% across all studies.^19^ The analysis showed that the largest decrease was observed in studies conducted in corticosteroid-naïve patients (a 93% reduction in BEC) compared to those including at least some patients on maintenance OCS treatment (a 60% reduction in BEC).^19^

In a study where all patients were on OCS maintenance therapy with at least 10 mg prednisolone daily for 6 months, the reduction in BEC was smaller (41%).^20^ It is important to highlight that in most evaluated studies, the mean BEC was lower than 150 cells/μL.

A post hoc analysis of the SIRIUS study, a randomized controlled trial involving patients with severe eosinophilic asthma to evaluate the corticosteroid-sparing effect of mepolizumab (an anti-interleukin 5, IL-5, monoclonal antibody), analyzed how different doses of OCS may impact BEC. All patients were treated with maintenance OCS (5–35 mg/day prednisone or equivalent) for 6 months, and, during the optimization phase (3–8 weeks), patients with controlled asthma tapered down the prednisone dose by 5 mg/day each week (if receiving 20–35 mg/day of prednisone) or 2.5 mg/day each week (if receiving 5–15 mg/day of prednisone). A regression analysis estimating changes in BEC following 1 mg/day and 5 mg/day decreases in prednisone dose showed that a 1 mg/day reduction resulted in a 7% increase in BEC. In contrast, a 5 mg/day reduction resulted in a 41% increase in BEC. In the 23% of patients who increased the OCS dose, there was a concomitant mean reduction of BEC of 130 cell/μL (corresponding to a 39% reduction), while those patients maintaining their OCS dose during the optimization phase achieved a mean reduction of BEC of 30 cells/μL (−12,5%).^21^

In a retrospective real-life study, asthmatic patients were divided into 3 subgroups based on BEC at baseline (≥150, ≥300, and ≥400 cells/μL) and treated with a dose of OCS of 35 mg/day for a median period of 3 weeks. After 19 days of treatment cessation, BEC was reduced by 112 (−30%), 202 (−34%), and 290 (−36%) cells/μL compared to the baseline, respectively. After 79 days, BEC was reduced by 65 cells/μL (−18%), 150 cells/μL (−26%), and 246 cells/μL (−32%) compared to the baseline, respectively. Therefore, BEC remained 32–177 cells/μL below baseline after a 79-day washout period.^12^

Finally, a recently published registry-based cohort study by Schleich et al, endorsed by the International Severe Asthma Registry (ISAR),^22^ involving 4305 patients with severe asthma, showed how the BEC varied according to OCS prescription patterns. Predictably, the BEC was lower in OCS-treated patients and even lower in those receiving long-term OCS compared to those receiving intermittent OCS, regardless of OCS dosage.

Interestingly, it has been shown that even with ICS at a medium to high dose, a significant reduction in BEC can be observed in patients with not well-controlled asthma.^23^ This raises concerns about how truly ‘normal’ the ‘normal values’ of BEC are in asthma patients, whether this reduction might prevent treatment with biologics targeting the interleukin-5 pathway, and the small but significant systemic absorption of steroids through ICS.^24^

In conclusion, these large studies demonstrate the impact of OCS on BEC, which is greater with a higher baseline level and a lower previous OCS exposure and more impactful with a long-term OCS course than an intermittent one. Although there is no conclusive data on the exact wash-out period needed to determine a reliable baseline BEC in patients treated with OCS, the literature suggests that 3 months could be a trustworthy value.

##### Serum total IgE and specific IgE

In the context of allergic respiratory diseases, serum total IgE (tIgE) is a biomarker of T-2 inflammation useful to phenotype severe allergic asthma and also used to make an alternative diagnosis like allergic bronchopulmonary aspergillosis (ABPA).^25^ In clinical practice, tIgE in asthmatic patients is also analyzed to estimate the optimal dosage of the anti-IgE antibody omalizumab for add-on treatment in severe allergic asthma.^18^ Specific serum IgE (sIgE) directed against allergen extracts is used to study sensitization to aeroallergens and to support the clinical relevance of allergic respiratory diseases, such as asthma.^26^

Although it is clear and predictable that OCS treatment impacts tIgE and sIgE, the exact extent of this influence is still subject to study. It has been observed that short-term treatment with OCS can induce a transient polyclonal IgE increase by 18% or 46% in asthmatic patients after 7 or at least 14 days of treatment with 20 mg prednisone, respectively. IgE levels returned to baseline or below 22 days after discontinuation of SCS.^27^, ^28^ Nonetheless, in another study, it has been demonstrated that, after a transient increase, if the treatment was ongoing for 6–8 weeks, there was a decrease in serum IgE with no difference compared to the pretreatment levels.^29^ It was also seen that prolonged treatment for several months can induce depression of the IgE level in asthmatic children compared to the control group. It must be considered that, in the study above, the treatment period wasn't the same for all patients.^30^ Another study demonstrated that sIgE were more readily inhibited than non-specifically induced IgE^31^ Accordingly, in the aforementioned ISAR study on patients with severe asthma,^22^ a reduction in tIgE concentration distribution has been shown in both long-term and intermittent OCS cohorts, with significantly lower concentrations observed in the first group.

About specific IgE, predictably, short treatment with OCS, like oral prednisone 20 mg twice a day for 1 week, can induce a polyclonal IgE response with an increase of sIgE levels to multiple perennial and seasonal allergens in asthmatic patients.^27^ Another study was conducted during the pollination season, in which patients with allergic rhinitis to ragweed pollen were divided into 2 groups: a control group (no treatment) and an intervention group treated with a single injection of 60 mg of triamcinolone acetonide, followed by 16 mg of methylprednisolone administered orally for 3 days. The result was that ragweed-specific IgE increased after pollen exposure in both groups, but there was no significant difference in the increases.^32^

The studies in the literature do not agree on the effect of OCS on the levels of tIgE and sIgE. No evidence for suppression of IgE levels that could lead to false-negative results in IgE measurements is reported. Therefore, there is no recommendation for discontinuing or adjusting the dosage of an ongoing medication with glucocorticoids before IgE measurement for diagnostic purposes. However, the abovementioned aspects should be considered when interpreting absolute levels of IgE detected under ongoing medication.

##### Precipitins

A precipitin is an antibody that can precipitate out of a solution upon binding to an antigen. Precipitin assays are commonly used to diagnose infectious diseases.

This test can be useful in diagnosing asthmatic patients with ABPA, a type I and III hypersensitivity reaction in response to colonization of the airways with *Aspergillus fumigatus*. There is no consensus on the exact set of diagnostic criteria for ABPA. Still, positive serum precipitins to Aspergillus fumigatus and a demonstration of hypersensitivity reactions to Aspergillus are included in most diagnostic guidelines.^33^

Precipitating antibodies or specific IgG to the crude antigen are used as an instrument to evaluate type III hypersensitivity to *A. fumigatus*. A lower percentage of non-specific precipitins may be due to patients on cortisone therapy. Still, the positive rate for *Aspergillus-specific* IgG in cases treated with or without systemic corticosteroids does not seem to differ.^34^ There is no more data available about the effect of steroids on precipitin serum levels.

#### *Clinical research*

##### Interleukin 4, 5, 13, 25, and 33, thymic stromal lymphopoietin, CCL-26, and CCL-17/TARC

Numerous cytokines are known to orchestrate the pathogenesis of asthma. The measurement of interleukins is not a routine test in the diagnostic and therapeutic evaluation of patients with asthma; it is currently reserved mainly for research purposes.^1^ The administration of OCS can potentially reduce cytokine serum levels due to its potent, broad anti-inflammatory effects.^35^, ^36^, ^37^, ^38^ Nevertheless, few measurements of this plausible reduction are available in the literature. OCS has been demonstrated to reduce the production of IL-5 both *in vitro*^39^ and *in vivo*^20^ (without normalizing the values in 1 report^40^ in some studies, but not in others.^41^, ^42^ Similar unconclusive results can be found in IL-4.^40^, ^43^ The effect of steroids on reducing the activity of IL-13 has been demonstrated in animal models^44^ and in a single study on severe OCS-dependent asthmatics.^20^ The same authors successfully measured the return of serum IL-5 and IL-13 to baseline values at 60 days, suggesting that waiting 1 month before using type-2 biomarkers to guide treatment in OCS-dependent asthmatics is warranted.^20^ Few data are available on the impact of steroids on serum alarmins. *In vitro*, CS suppresses the dsRNA-induced release of TSLP from keratinocytes in an atopic cytokine milieu^45^ Nonetheless, the TSLP/innate lymphoid cells axis may mediate steroid resistance in asthma.^46^, ^47^ Therefore, the drug's effect on serum TSLP concentrations may not be predictable. One single study demonstrated that 0.5 mg/kg/day of oral prednisolone for 14 days reduced the serum level of CCL17 in asthmatic patients.^48^ Finally, there is no available data on the impact of OCS on serum levels of IL-25 and IL-33.

##### Dipeptidyl peptidase-4 (DPP-4)

Dipeptidyl peptidase-4 (DPP-4) is a glycoprotein expressed on the cell surface of immune cells. DPP-4 has a broad spectrum of biological functions, including immune regulation and glucose metabolism. DPP-4 may be a novel biomarker for diagnosing and determining disease severity, as well as monitoring treatment response, in asthmatic patients.^49^ The levels of DPP-4 are significantly higher in patients with both acute and chronic asthma compared to healthy controls.^50^ Treatment with ICS ± bronchodilators and montelukast is associated with a significant decline in serum DPP-4 levels in asthma patients in both acute and chronic settings,^50^ with no data available in the literature regarding OCS.

##### Eosinophil cationic protein (ECP)

Eosinophil cationic protein (ECP), also known as ribonuclease 3 (RNAse3), is one of the most toxic granulocyte-derived proteins released by activated eosinophils in inflammatory conditions and is a marker of eosinophil activation in the body. It is a potent cytotoxic molecule with a possible role in tissue remodeling, altering pulmonary surfactant structure and function, and contributing to airway obstruction.^50^

The levels of ECP in serum and sputum samples have been shown to correlate with the severity of asthma.^51^ Steroid treatment can reduce both the number of eosinophils and, consequently, the levels of ECP, which are markers of inflammation and tissue damage in the airways of asthma patients.^52^

##### Periostin

Periostin is a systemic biomarker of airway eosinophilia in asthmatic patients and has potential utility in patient selection for emerging asthma therapeutics targeting Th2 inflammation.^53^ Periostin enhances profibrotic TGF-β signaling in subepithelial fibrosis associated with asthma. IL-13 induces bronchial epithelial cells to secrete periostin. IL-13 levels in sputum and bronchial biopsy samples remain elevated in severe asthma, despite the use of inhaled and systemic corticosteroids.^54^ There is no available data on the impact of OCS.

### Instrumental biomarkers

The OCS's impact on humoral biomarkers will be reviewed in the following paragraphs, divided into biomarkers used in clinical practice and research settings. The data collected are summarized in Table 3.

**Table 3:** **Instrumental biomarkers and relationship with systemic corticosteroid therapy. References in text.**ABG: Arterial Blood Gas; BDR: Bronchodilator test; CT: Computed Tomography; DLCO: Diffusing capacity of the lungs for carbon monoxide; ECB: Exhaled Breath Condensed; FeNO: Fraction of Exhaled Nitric Oxide; HRTC: High-resolution Computed Tomography; ICS: inhalant corticosteroids; INCS: intranasal corticosteroids; IOS: Impulse oscillometry; MCS: Mycobacteria sp.; MCT: Metacholine Challenge Testing; PSG: Polysomnography; SPT: Skin Prick Test; WBP: Whole Body Plethysmography

Instrumental biomarker	Application in clinical practice	Altered by steroids	Possible adjustment
**Spirometry**	Assess dynamic lung volumes.	Risk of false negative results	If needed, perform the test away from the OCS course.
**BDR**	Diagnostic methods in obstructive disorders	No/a little	If needed, perform the test before starting OCS
**IOS**	Measure airway resistance and lung elasticity	Risk of false negative results	If needed, perform the test away from the OCS course.
**WBP**	To measure static lung volumes	Risk of altering the results	If needed, perform the test away from the OCS course.
**FeNO**	Evaluate airway inflammation	Decreased according to most studies	Perform the test away from the OCS course
**MCT**	Asthma diagnosis	Risk of false negative results	Perform the test before starting the OCS course
**Chest X-Ray**	Diagnosis of infectious events during asthma exacerbations	It can make differential diagnosis difficult	N/A
**Chest HRCT** **And mucus plug score**	Assess pulmonary impairment, bronchiectasies, inflammatory foci, and mucus plugs	It can make differential diagnosis difficult	If possible, perform the test before starting OCS
**Maxillofacial CT**	Evaluation of asthma comorbidities like CRSwNP	It can mask the true severity of the chronic condition	If possible, perform the test before starting OCS
**Nasal citology**	Analysis of nasal mucosal cells	It can make results unreliable	Stop CS treatment almost 7 days before the test
**Induced sputum**	Analysis of cells in sputum	Risk of false negative results	Perform the test before starting OCS
**SPT**	Test allergen sensitization	Risk of false negative results	Suspend OCS 72 h before the test
**ABG**	Assessment of blood pH and dissolved gas	N/A	N/A
**Sputum culture**	Evaluation of possible infection	Probable, with unclear outcomes	Perform the test before starting OCS
**MCS/Atypical**	Evaluation of possible infection	N/A	N/A
**ECB**	Unclear	Reduced by ICS (no data on OCS)	N/A
**Multi-flow exhaled nitric oxide and nasal nitric oxide**	Unclear	Decreased by ICS and INCS	Perform the test away from the OCS course

#### Clinical practice

##### Spirometry

Spirometry is the most important instrumental test for evaluating the asthmatic patient in both the acute and chronic phases, and it is a fundamental tool in managing asthma as a whole, from diagnosis to evaluation of the response to therapy and assessment.^1^ OCS can improve spirometry parameters in patients with significant bronchial inflammation, such as in severe asthma and some forms of COPD with an eosinophilic component.^1^, ^55^ A meta-analysis about the effect of ICS on lung functions in patients affected by asthma or COPD showed that 600 μg of fluticasone can induce a change in FEV1 of a mean of 710 ml,^56^ while another one showed that a course of OCS of 40 mg could improve the FEV1 of 9% compared with the baseline.^19^ While a short-term course of OCS can modestly improve spirometry values in patients with chronic obstructive pulmonary disease (COPD), it is unknown whether this improvement is sustained with chronic OCS treatment.^57^ A trial on prolonged OCS therapy showed that the treatment of a low dose (5 mg) daily prednisolone alongside inhaled corticosteroids in patients with moderate COPD did not yield further enhancement in pulmonary function.^58^ Thus, there is no clear indication for CS withdrawal before performing forced spirometry.

##### Bronchodilator reversibility test

The bronchodilator test (BDR) offers an important added value, as it allows for the assessment of reversibility of respiratory changes. Based on the degree of reversibility observed after the use of a short-acting beta agonist (SABA), a diagnosis of asthma can be made.^59^ Inhaled corticosteroids should not be withheld before a bronchodilator reversibility test.^60^ A study showed that short-term treatment with OCS does not modify the bronchodilator reversibility test.^61^ At the current time, there is no indication to withhold CS before BDR.

##### Impulse oscillometry

Impulse oscillometry (IOS) allows passive assessment of lung mechanics by superimposing sound waves onto normal breathing. It analyses flow and pressure variations to measure airway resistance and lung elasticity.^62^ The use of OCS can impact IOS in several ways, primarily by reducing inflammation and improving airway patency.^63^ In particular, a study showed how 4 weeks of ICS (eg, budesonide) could reduce small airway resistance parameters R5 and R20 by a mean of −0.06 kPaL^−1^·s.^64^

##### Whole body plethysmography

Whole Body Plethysmography (WBP) is a technique that measures static lung volumes. The parameters measured are Residual Functional Capacity (FRC) and airway resistance (Raw). The FRC allows to calculate Total Lung Capacity (TLC) and Residual Volume (RV).^65^ The use of OCS in plethysmography shows similar effects to those observed in spirometry and IOS, with some peculiarities: decrease in RV and FRC and reduction in hyperinflation; decrease in airway resistance (Raw) and increasing by double the specific conductance (sGaw^55^, ^66^, ^67^).

##### Fractional exhaled nitric oxide (FeNO) 50 ml/s

Nitric oxide (NO) is physiologically present in the lungs of healthy people at low concentrations. In T2 asthmatic patients, the concentration of NO is increased, leading to airway hyperresponsiveness. Cellular damage, eosinophilia, mucous hypersecretion, and increased vascular permeability, reflecting IL-13 and IL-4 activity.^68^ Fractional exhaled nitric oxide (FeNO) is recognized as a non-invasive, safe, and repeatable biomarker of type 2 airway inflammation. Its accuracy as a surrogate marker of sputum eosinophilia is similar to serum eosinophilia and greater than total serum IgE. Furthermore, compared with serum eosinophilia, which originates from the systemic circulation and may reflect the conditions of other patients, FeNO directly correlates with T2 inflammation in the airways.^69^ FeNO predicts responsiveness to ICS and aids in determining the optimal dosage for asthma management. Moreover, FeNO may assist in selecting treatment with monoclonal antibodies for asthma. The use of CS leads to a significant reduction in FeNO (50 mL/s) in patients with eosinophilic asthma, as it inhibits iNOS expression, resulting in a decrease in NO production.^70^ A trial involving a pediatric population of 92 asthmatic patients demonstrated that a short course of prednisone (5–7 days) reduces FeNO values by a mean of 56.6%.^71^ Interestingly, the aforementioned ISAR study found less evidence of the OCS masking effect on FeNO.^22^ There are indications to withhold inhaled steroids a week before performing the FeNO measurement.^72^

##### Methacholine challenge testing

Methacholine challenge testing (MCT) is a common form of bronchoprovocation, using the longer-acting acetylcholine derivative methacholine to induce bronchoconstriction. High-dose prednisone therapy (60 mg/day) can also reduce bronchial sensitivity to methacholine, improving baseline FEV1 and FEF25-75 in asthmatic children.^73^, ^74^ There is no need for withholding inhaled corticosteroids because they have little or no effect on MCT.^75^ There are no clear indications on when to withhold OCS before performing MCT.

##### Chest X-ray, maxillofacial CT

Chest X-ray is a common first-line radiological examination for bronchial asthma, especially during infectious events. Maxillofacial CT evaluates conditions associated with bronchial asthma. Corticosteroid use can affect bone density and tissue structure, potentially altering radiological appearances.^76^, ^77^

##### High resolution computed tomography (HRTC) and CT mucus score

High resolution computed tomography (HRCT) could help to distinguish between pathologies with similar clinical presentation but different radiologic patterns, eg, severe asthma and Eosinophilic Granulomatosis with Polyangiitis (EGPA).^78^ OCSs appears to reverse some of the asthma-induced structural modifications (airway remodeling), particularly the greater vascularity of the bronchial wall.^79^ Furthermore, a 2012 study performed on asthmatic patients showed how inhaled and systemic CSs could have an impact on small airways, reducing air trapping.^80^ Mucus plugs are found in CT scans of more than a half of patients affected by severe asthma.^81^ Apparently inhaled or systemic CSs have no effect on mucus score as evaluated per CT scans^82^

##### Nasal cytology

Nasal cytology is a technique used to analyze nasal mucosal cells and evaluate the effects of treatments.^83^ OCS reduce primarily eosinophils in nasal samples, while intranasal corticosteroids (INCS) such as mometasone and fluticasone notably decrease both eosinophils and neutrophils.^84^, ^85^, ^86^, ^87^, ^88^ Mometasone may also impact the bacterial biofilm in patients with chronic rhinosinusitis with nasal polyps (CRSwNP).^89^ Cellular infiltration typically returns within 3–6 days after stopping CS,^90^ so we could consider a minimal washout of 7 days for both OCS and INCS therapy before performing nasal cytology.

##### Induced sputum

Cell analysis in induced sputum is a safe and non-invasive method for evaluating airway inflammation.^91^ Oral prednisone 0.5 mg/kg/day reduces sputum eosinophils and ECP within 2–7 days,^92^, ^93^ Inhaled corticosteroids, used for over 6 weeks for a cumulative dose >60 mg/day, significantly reduce neutrophil counts and inflammatory markers in sputum.^94^ Since there are no clear guidelines regarding the duration of OCS suspension before induced sputum, assessing it before the molecule has been administered is recommended.

##### Skin prick test

The Skin Prick Test (SPT) is a safe, quick, and effective method for testing a patient's allergen sensitivities. Predictably, OCS and the application of topical dermal corticosteroids can affect SPT outcomes. Suspension for 72 h for all steroid treatment is recommended.^95^, ^96^

##### Arterial blood gas

Currently, no studies have demonstrated significant other alterations in arterial blood gas analysis in patients using steroids.

##### Sputum culture

Studies on asthma patients reveal a high presence of bacteria like *Haemophilus influenzae*, *Moraxella catarrhalis*, and *Tropheryma whipplei*, with a positive correlation with severe asthma and eosinophil detection, suggesting that antibiotic therapies and the use of oral corticosteroids can modify the microbiota.^97^, ^98^

##### Search for mycobacteria and atypical bacteria

The use of inhaled corticosteroids (ICS), particularly in patients with asthma and COPD, may increase the risk of tuberculosis infection.^99^ Currently, there is no data in the literature on how OCS treatment may alter the ability of common tests to detect atypical bacteria and/or mycobacteria in sputum.

#### Clinical research

##### Exhaled breath condensed (EBC)

Exhaled breath condensate (EBC) analysis is a non-invasive procedure for identifying biomarkers originating from the lower respiratory tract, obtained by cooling and condensing exhaled aerosol.^100^ A study by Carpagnano et al on EBD showed that the pH and IL-4 levels were significantly reduced after 6 months of ICS treatment, whereas IL-6 levels did not change from baseline.^101^ Nonetheless, there is no data about the impact of OCS on this biomarker.

##### Multiple flow exhaled nitric oxide

The change in FeNO, measured at various standardised controlled flow rates, may provide more insights than normal measures at 50 ml/s,^102^ permitting the measurement of CalvNO and J'awNO, respectively, the alveolar and bronchial component of FeNO.^103^ Despite ICS use being expected to reduce exhaled NO, 1 study on the pediatric population showed that subjects treated with ICS more recently had a high value of FeNO at 50 ml of flux and at bronchial flux (J'awNO), probably reflecting the severity of their asthma.^102^ Similar results were found in the adult population.^104^ As stated for FeNO, withholding ICS for a week appears to be a correct management approach.^72^

##### Nasal nitric oxide (nNO)

Nasal nitric oxide (nNO) is the fraction of nitric monoxide produced by the nasal and paranasal sinus mucosa and reflects the inflammation of the upper airway. Two studies showed how nNO decreases significantly after a prolonged treatment with INCS (eg, budesonide).^105^, ^106^

### Clinical biomarkers

The OCS's impact on clinical biomarkers and evaluation will be reviewed in the following paragraphs.

#### Ear-nose-throat examination

The most common comorbidities of asthma patients that may require specialized ear, nose, and throat (ENT) expertise are chronic rhinosinusitis with or without nasal polyps.^107^, ^108^, ^109^ In chronic rhinosinusitis, the use of steroids is fundamental. It is not uncommon to administer a short course of OCS in patients with severe CRSwNP due to easy availability, low cost, rapid action, and great effectiveness in basically all patients.^110^ The data on the efficacy of OCS on CRS without NP are less strong.^111^ Even a brief cycle of OCS can affect the ENT evaluation, leading to a significant improvement in every clinical dominium reported by patients (improving nasal congestion, smell dysfunction and other sinonasal symptoms)^52^, ^111^, ^112^ and in objective parameters (drastic reduction of mucosal oedema^113^, ^114^ and polyps’ number and dimension^52^, ^111^). This improvement may last several weeks after cessation of therapy,^52^ but will eventually fade. Due to the benefits being merely temporary, the risks of adverse reactions, cumulative side effects, and the onset of steroid-related disorders significantly overshadow the benefits of the chronic use of OCS.^115^, ^116^, ^117^

##### Gastroenterological examination

Asthma and gastrointestinal disorders may be interconnected in some circumstances, particularly when dealing with syndromes such as gastroesophageal reflux-induced asthma (GERD). GERD and asthma had a bidirectional relationship.^118^

Recent investigations on esophageal function and tissue biomarkers in patients with asthma and associated GERD have established a relevant role for esophageal motility and neuronal sensory abnormalities in linking the 2 diseases. Characterization of the underlying inflammatory substrate has yielded mixed results, as both neutrophilic and eosinophilic type 2 inflammatory changes have been described.^119^

GERD can lead to more frequent exacerbations in asthma patients, and dealing with this comorbidity effectively can help reduce the risk of asthma exacerbations.^120^, ^121^ The management of this condition is complex and necessitates the use of various synergistic strategies, which usually do not include OCS. Moreover, steroid therapy does not significantly alter the mucosal lesions detectable in patients with GERD.^122^

## Conclusion

**Fig. 1:**
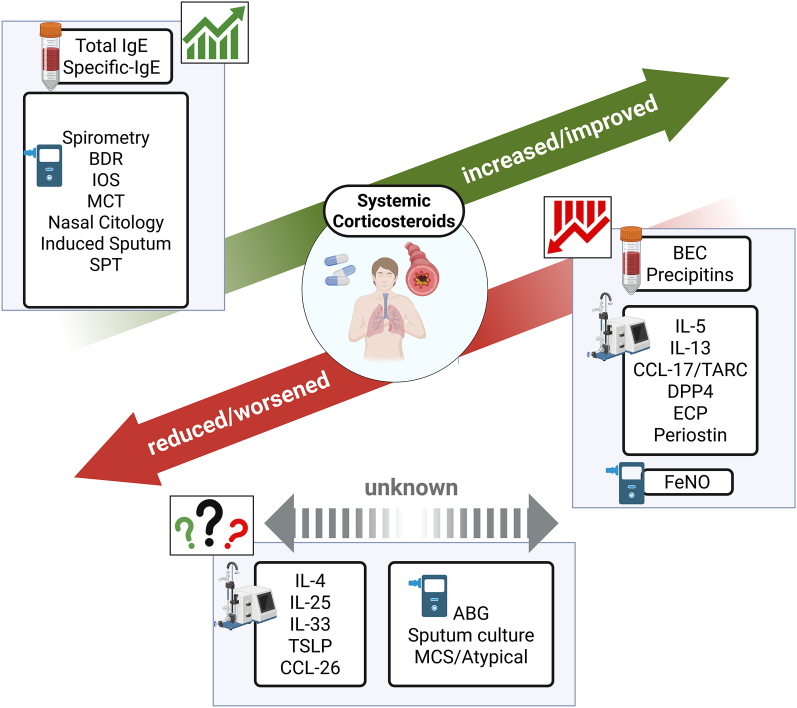
This figure illustrates how the use of corticosteroids (CS) in asthmatic patients can affect certain disease biomarkers. Specifically, the green arrow shows biomarker values increased or improved by CS, potentially leading to a better outcome than would be obtained without CS (risk of false positives). The red arrow indicates the parameters that are reduced by CS, potentially masking a pathological value (risk of false negative). The dashed grey arrow indicates that there are no data on the impact that CS has on those biomarkers. ABG: Arterial Blood Gas; BEC: Blood Eosinophil Count; BDR: Bronchodilator Response; CCL-TARC: Chemokine (C–C motif) Ligand - Thymus and activation-regulated chemokine; DPP4: Dipeptidyl Peptidase 4; ECP: Eosinophil Cationic Protein; FeNO: Fractional exhaled Nitric Oxide; IgE: Immunoglobulin E; IL: Interleukin; IOS: Impulse oscillometry; MCS: Mycobacteria sp; MCT: Methacholine Challenge Test; SPT: Skin Prick Test; TSLP: Thymic Stromal Lymphopoietin

Systemic corticosteroids remain a drug class currently utilized in clinical practice for managing asthma, particularly in severe cases and during acute episodes of exacerbation. Their ability to rapidly suppress inflammation and restore airway function is invaluable, with both short-term and long-term side effects. Their widespread use presents significant challenges, particularly in interpreting asthma biomarkers. As this analysis has demonstrated, systemic steroids can profoundly alter clinical, humoral, and instrumental biomarkers, often leading to an overestimation of disease control and a misrepresentation of the underlying inflammatory burden [Fig. 1].

One of the most concerning aspects of systemic steroid use is its potential to create a “cosmetic” improvement in biomarker profiles, temporarily normalizing values such as eosinophil counts, FeNO levels, and cytokine expression. This can obscure the true severity of asthma and complicate long-term management, leading to underestimation of persistent inflammation, inappropriate step-down therapy, or failure to recognize steroid dependence. Furthermore, the widespread, and often undocumented, overuse of systemic steroids—either self-prescribed or administered without adequate tracking—further exacerbates this issue, making it even more challenging to phenotype patients and tailor treatment strategies accordingly accurately.

Given these challenges, it is crucial to develop and implement strategies that allow for a more accurate interpretation of biomarker data in patients undergoing systemic steroid therapy. Several approaches may help mitigate the confounding effects of corticosteroids, including the use of correction factors, adjusted interpretation thresholds, and the integration of multi-modal biomarker assessments.

Additionally, there is an urgent need for more robust correction tools that account for the pharmacodynamic effects of systemic steroids. Research should focus on developing predictive models that can adjust biomarker values based on steroid dosage, duration, and individual patient response. This would enable clinicians to distinguish true disease remission from steroid-induced suppression of inflammatory markers, ultimately leading to more precise asthma phenotyping and better long-term outcomes.

Future studies should also explore alternative biomarkers that are less susceptible to steroid interference. While traditional markers such as blood eosinophils and FeNO are well-established, emerging biomarkers—including periostin, DPP-4, and specific cytokine profiles—may offer additional insights into steroid-resistant inflammation and asthma endotypes. A more nuanced understanding of how systemic steroids impact different biomarker pathways will be key to refining asthma management in the era of precision medicine.

Ultimately, the findings of this comprehensive analysis underscore the importance of cautious interpretation of biomarker data in patients treated with steroids. The influence of systemic corticosteroids on disease assessment must be carefully considered to avoid misclassification and suboptimal treatment decisions. Moving forward, a combination of improved biomarker correction strategies, better patient education on steroid use, and ongoing research into novel inflammatory markers will be essential in enhancing the accuracy and effectiveness of asthma management. By addressing these challenges, we can take a significant step toward more individualized, evidence-based care for patients with asthma.

## Authorship

All authors have made substantial contributions to the conception and design of the study, drafting the article, and revising it critically for important intellectual content. All authors approved the version submitted.

## Declaration of generative AI and AI-assisted technologies in the writing process

Nothing to disclose.

## Financial disclosure

No funds were received for this study.

## Competing interests

Giovanni Paoletti reports fees for speaker activities and/or advisory boards participation from Lofarma, GSK, and AstraZeneca, outside the submitted work. Giorgio Walter Canonica reports research or clinical trials grants paid to his Institution from Menarini, AstraZeneca,GSK, Sanofi Genzyme and fees for lectures or advisory board participation from Menarini, AstraZeneca, CellTrion, Chiesi, Faes Farma, Firma, Genentech, Guidotti-Malesci, GSK, HAL Allergy, Innovacaremd, Novartis, OM-Pharma, Red Maple, Sanofi-Aventis, Sanofi-Genzyme, Stallergenes-Greer and Uriach Pharma, outside the submitted work. Enrico Heffler reports fees for speaker activities and/or advisory boards participation from Sanofi, Regeneron, GSK, Novartis, AstraZeneca, Stallergenes-Greer, Chiesi, Almirall, Bosch, Lofarma, outside the sumitted work. Stefano Del Giacco has received speaker fees by AstraZeneca, Chiesi, GSK, Guidotti, Menarini, Novartis, Sanofi, Stallergenes, Takeda, Celltrion and Valeas. He has received advisory board fees from AstraZeneca, Chiesi, CSL-Behring, GSK, Novartis, Sanofi and Takeda, and has received research grants from AstraZeneca, CSL.Behring, GSK, Sanofi and Novartis. Giovanni Costanzo, Marta Marchetti, Guido Valentini, Alfredo Scardini, Giada Sambugaro, Martina Bullita, Sofia Vassallo, Andrea Giovanni Ledda, Davide Firinu, Giulia Anna Maria Luigia Costanzo report no conflicts of interest.
